# ﻿Two new *Clitocella* species from North China revealed by phylogenetic analyses and morphological characters

**DOI:** 10.3897/mycokeys.88.80068

**Published:** 2022-04-13

**Authors:** Ning Mao, Jing-Chong Lv, Yu-Yan Xu, Tao-Yu Zhao, Li Fan

**Affiliations:** 1 College of Life Science, Capital Normal University, Xisanhuanbeilu 105, Haidian, Beijing 100048, China Capital Normal University Beijing China

**Keywords:** Entolomataceae, multigene, phylogeny, taxonomy

## Abstract

Two new species of *Clitocella* are proposed based on morphological and phylogenetic investigations. *Clitocellaborealichinensis***sp. nov.** is closely related to *C.orientalis* but distinguished from the latter by its slightly smaller basidiospores and hyphae of pileipellis with pale brown to brown intracellular or parietal pigment. *Clitocellacolorata***sp. nov.** is closely related to *C.popinalis* and *C.mundula* in macromorphology but is differentiated from *C.popinalis* by its slightly smaller basidiospores and the difference in genetic profile, and from *C.mundula* by its relatively colorful pileus (white to yellowish white, grayish white to grayish brown, pink white). Phylogenetic analyses based on sequence data from five different loci (ITS, nrLSU, *tef1*, *rpb2* and *atp6*) support the taxonomic position of the two new species in the genus *Clitocella*. The illustrations and descriptions for the new taxa are provided.

## ﻿Introduction

The genus *Clitocella* Kluting, T.J. Baroni & Bergemann (Entolomataceae, *Agaricales*), with *C.popinalis* (Fr.) Kluting, T.J. Baroni & Bergemann as the type species, was established in 2014 (Kluting et al. 2014). The main characteristics of *Clitocella* are clitocyboid basidiomata, narrow and crowded, long-decurrent lamellae, central to eccentric stipe, thin-walled (<0.5 μm) basidiospores with undulate pustules or minute bumps, clamp connections absent. (Baroni 1981; Kluting et al. 2014; Jian et al. 2020). Previous studies show that *Clitocella* is phylogenetically closely related to the genera *Clitopilus* (Fr. ex Rabenh.) P. Kumm. and *Clitopilopsis* Maire. *Clitopilus* differs from *Clitocella* in its longitudinally ridged basidiospore ornamentation, and *Clitopilopsis* in its basidiospores with thickened walls (0.5–0.9 μm) and obscure irregular rounded angles of the basidiospores in polar view (Kluting et al. 2014; Baroni et al. 2020; Jian et al. 2020). There are 10 accepted species in *Clitocella* (Index Fungorum, http://www.Indexfungorum.org/; accessed date: 19 November 2021).

In China, the species diversity of *Clitocella* is scarce and only two species are described (Jian et al. 2020). Recently, several specimens of *Clitocella* were collected when we investigated the macrofungi in Shanxi province, North China. The morphological examination and phylogenetic analysis for these collections revealed that they represented three taxa of *Clitocella*, including two new species. The aim of this paper is to describe the new species and provide the DNA data to confirm the presence in China of a previously described species.

## ﻿Materials and methods

### ﻿Morphological studies

Collections were obtained and photographed in the field from Shanxi regions in China, and then dried in a fruit drier at 40–50 °C and deposited in BJTC herbarium (Capital Normal University, Beijing, China). The sizes of basidiomata (pileal width) used in this study are as follows: small: <30 mm; medium-sized: 30–50 mm; large: >50 mm. Standardised color values were obtained from ColorHexa (http://www.colorhexa.com/). Microscopic characters were observed in sections obtained from dry specimens mounted in 3% KOH, Congo Red, or Melzer’s reagent (Dring 1971). For scanning electron microscopy (SEM), basidiospores were scraped from the dried gleba, placed onto double-sided tape that was mounted directly on the SEM stub, coated with platinum-palladium film of 8 nm thick using an ion-sputter coater (HITACHI E-1010), and examined with a HITACHI S-4800 SEM. The term “[n/m/p]” means n basidiospores from m basidiomata of p collections. Dimensions of basidiospores are given using the following format ‘(a–)b–c(–d)’, where the range ‘b–c’ represents at least 90% of the measured values, and ‘a’ and ‘d’ are the most extreme values. L_m_ and W_m_ indicate the average basidiospore length and width (± standard deviation) for the measured basidiospore, respectively. ‘Q’ refers to the length/width ratio of basidiospores in side-view; ‘Q_av_’ refers to the average Q of all basidiospores ±standard deviation.

### ﻿DNA extraction, PCR amplification and DNA sequencing

Dried basidiomata were crushed by shaking for 45 s at 30 Hz 2–4 times (Mixer Mill MM301, Retsch, Haan, Germany) in a 1.5 mL tube together with a 3 mm diam tungsten carbide ball. Total genomic DNA was extracted from the powdered basidiomata using NuClean Plant Genomic DNA Kit (CWBIO, China), following the manufacturer’s instructions. Primers ITS1F and ITS4 were employed for the ITS (White et al. 1990; Gardes and Bruns 1993), while LR0R and LR5 for nrLSU (Vilgalys and Hester 1990), EF1-983F and EF1-1953R for the *tef1* (Rehner 2001), bRPB2-6F and bRPB2-7R2 for the *rpb2* (Liu et al. 1999; Matheny 2005; Matheny et al. 2007), and ATP6-3 and ATP6-6r for the *atp6* (Kretzer and Bruns 1999; Binder and Hibbett 2003). Polymerase chain reactions (PCR) for ITS region, nrLSU region, *tef1* gene, *rpb2* gene and *atp6* gene were performed in 25 µL reaction containing 2 µL DNA template, 1 µL primer (10 µM) each, 12.5 µL of 2× Master Mix [Tiangen Biotech (Beijing) Co.], 8.5 µL ddH_2_O.

PCR reactions were implemented as follows: an initial denaturation at 94 °C for 5 min, then to 35 cycles of the following denaturation at 94 °C for 30 s, annealing at 52 °C for 45 s (ITS), 60 s (nrLSU), 72 °C for 1 min; and a final extension at 72 °C for 10 min. Amplification of *rpb2* and *tef1* sequences followed Kluting et al. (2014), which entailed a touchdown protocol: an initial incubation of 94 °C for 5 min; 12 cycles of 94 °C for 1 min, 67 °C for 1 min, decreasing 1 °C each cycle, and 72 °C for 1.5 min; 36 cycles of 94 °C for 45 s, 55 °C for 1 min, and 72 °C for 1.5 min; and a final extension period at 72 °C for 7 min. Sequences of the *atp6* were amplified with a cycling protocol of 95 °C for 5 min, followed by 40 cycles at 95 °C for 30 s, 42 °C for 2 min, and 72 °C for 1 min, and a final extension at 72 °C for 10 min. The PCR products were sent to Beijing Zhongkexilin Biotechnology Co. Ltd. for purification, sequencing, and editing. Validated sequences were deposited in the NCBI database (http://www.ncbi.nlm.nih.gov/). Other sequences of *Clitocella* and related species were mainly selected from those used by previous studies (Kluting et al. 2014; Vizzini et al. 2016; Baroni et al. 2020; Jian et al. 2020). The accession numbers of all sequences employed are provided in Table 1.

**Table 1.** Specimens used in molecular phylogenetic studies and their GenBank accession numbers. Newly generated sequences are in bold.

Species	Voucher	Locality	GenBank accession No.				
			ITS	nrLSU	*rpb2*	*tef1*	*atp6*
*Catathelasmaventricosum*	DAOM221514	USA	KP255469	–	–	–	–
***Clitocellacolorata***	**BJTC FM1593**	**China**	**OL966940**	–	–	–	–
***Clitocellacolorata***	**BJTC FM1594**	**China**	**OL966941**	–	–	–	–
***Clitocellacolorata***	**BJTC FM1891**	**China**	**OL966944**	**OL966955**	**OL989914**	**OL989918**	**OL989924**
***Clitocellacolorata***	**BJTC FM1892**	**China**	**OL966945**	**OL966956**	**OL989915**	**OL989919**	**OL989925**
***Clitocellacolorata***	**BJTC FM1952**	**China**	–	**OL966958**	**OL989916**	**OL989920**	**OL989926**
*Clitocellafallax*	CBS 605.79	–	AF357018	–	–	–	–
*Clitocellafallax*	CBS 129.63	–	AF357017	AF223166	EF421018	–	–
*Clitocellafallax*	K(M): 116541	Spain	–	–	KC816938	KC816847	KC816769
*Clitocellafallax*	O-F88953	Norway	–	–	KC816936	KC816845	KC816767
*Clitocellafallax*	25668OKM	USA	–	–	KC816937	KC816846	KC816768
*Clitocellafallax*	ME Noordeloos 1997173	Italy	–	GQ289209	GQ289275	–	–
*Clitocellafallax*	ME Noordeloos 200367	Slovakia	–	GQ289210	GQ289276	–	–
*Clitocellamundula*	7161 TJB	USA	–	–	KC816952	KC816862	KC816782
‘*Clitocellamundula*’	O-F19454	Norway	–	–	KC816954	KC816864	KC816784
*Clitocellamundula*	O-F71544	Norway	–	–	KC816950	KC816860	KC816780
‘*Clitocellamundula*’	AFTOL-ID 521	USA	–	–	KC816953	KC816863	KC816783
*Clitocellamundula*	7115 TJB	USA	–	–	KC816951	KC816861	KC816781
*Clitocellamundula*	K(M): 164736	UK	–	–	KC816949	KC816859	KC816779
‘*Clitocellamundula*’	K(M): 49620	UK	–	–	KC816948	KC816858	KC816778
*Clitocellamundula*	HMJAU 7274	China	–	MN065724	MN148161	MN166272	MN133781
*Clitocellamundula*	HMJAU 7275	China	–	MN065723	MN148160	MN166271	MN133780
*Clitocellamundula*	HMJAU 27014	China	–	MN065722	MN148159	MN166270	MN133779
***Clitocellaborealichinensis***	**BJTC FM1618**	**China**	**OL966942**	**OL966946**	**OL989912**	–	**OL989922**
***Clitocellaborealichinensis***	**BJTC FM1781**	**China**	**OL966943**	**OL966957**	**OL989913**	**OL989917**	**OL989923**
*Clitocellaorientalis*	HKAS 75548	China	MN061333	MN065727	MN148164	MN166275	MN133784
*Clitocellaorientalis*	HKAS 75664	China	MN061332	MN065726	MN148163	MN166274	MN133783
*Clitocellaorientalis*	HKAS 77899	China	–	MN065725	MN148162	MN166273	MN133782
*Clitocellaorientalis*	HKAS 78876	China	MN061334	MN065729	MN148166	MN166277	MN133786
*Clitocellaorientalis* (Holotype)	HKAS 78763	China	–	MN065728	MN148165	MN166276	MN133785
***Clitocellaorientalis***	**BJTC FM1539**	**China**	–	**OL966947**	**OL989911**	**OL989921**	–
*Clitocellapopinalis*	HBJU-550	India	KU561066	–	–	–	–
*Clitocellapopinalis*	CBS 481.50	UK	FJ770397	–	–	–	–
*Clitocellapopinalis*	KA12-1717	Korea	KR673647	–	–	–	–
*Clitocellapopinalis*	RA802-3b	USA	MK217434	–	–	–	–
*Clitocellapopinalis*	Smith-2018 iNaturalist # 17340579	USA	MK573922	–	–	–	–
*Clitocellapopinalis*	K(M): 143166	UK	–	–	KC816971	KC816878	KC816796
*Clitocellapopinalis*	K(M): 167017	UK	–	–	KC816972	KC816879	KC816797
*Clitocellapopinalis*	O-F63376	Norway	–	–	KC816974	KC816880	KC816799
*Clitocellapopinalis*	6378 TJB	Switzerland	–	–	KC816976	KC816882	KC816801
*Clitocellapopinalis*	O-F105360	Norway	–	–	KC816975	KC816881	KC816800
*Clitocellapopinalis*	K(M): 146162	UK	–	–	KC816970	KC816877	KC816795
‘*Clitocellapopinalis*’	MC2-TRENT	Italy	–	–	KC816973	–	KC816798
‘*Clitocellapopinalis*’	ME Noordeloos 9867	Austria	–	GQ289213	GQ289280	–	–
*Clitocellapopinalis*	TB6378	USA	–	AF261285	GU384654	–	–
*Clitocella. Mundula*	HMJAU 7275	China	MN061331	–	–	–	–
*Clitocellaobscura*	MK09051302	Czech Republic	KX271753	–	–	–	–
*Clitocellaprunulus*	G.v. Zanen F96065	–	KC885965	–	–	–	–
*Clitocella_termitophila*	CORT014751	Dominican Republic	–	–	MN893319	–	–
*Clitopilusbrunneiceps* (Holotype)	HKAS 104510	China	–	MN065684	MN148123	MN166234	MN133737
*Clitopilusyunnanensis* (Holotype)	HKAS 104518	China	–	MN065698	MN148136	MN166247	MN133752
*Clitopilus. Amarus*	A. d. Haan 98031	–	KC885963	–	–	–	–
*Cltopilopsisalbida* (Holotype)	HKAS 104519	China	–	MN065730	MN148167	MN166278	MN133787
*Lyophyllumdecastes*	Sundberg091007a	Japan	HM572548	–	–	–	–
*Mycenapura*	CBH371	Denmark	KF913023	–	–	–	–
*Rhodocybemellea*	CORT013885	Dominican Republic	MN784992	–	–	–	–
*Rhodocybemellea*	JBSD127402	Dominican Republic	MN784993	–	–	–	–
*Rhodocybemellea*	CORT014470	Belize	MN784994	–	–	–	–
*Rhodocybemellea*	NYBG815044	Costa Rica	MN784995	–	–	–	–

### ﻿Phylogenetic analyses

The combined nrLSU-*rpb2*-*tef1*-*atp6* dataset and ITS dataset were compiled to identify new species and to investigate their phylogenetic position in *Clitocella*. For the combined nrLSU-*rpb2*-*tef1*-*atp6* dataset, *Clitopilopsisalbida* S.P. Jian & Zhu L. Yang was chosen as outgroups for individual (nrLSU, *rpb2*, *tef1*, *atp6*) or combined analyses (Jian et al. 2020). For ITS dataset *Mycenapura* (Pers.) P. Kumm. was selected as outgroup taxon (Baroni et al. 2020). The sequences of each marker (ITS, nrLSU, *rpb2*, *tef1*, *atp6*) were independently aligned in MAFFT v.7.110 (Katoh and Standley 2013) under default parameters. Ambiguously aligned sites were identified by Gblocks v.0.91b (Castresana 2000; using default options except “Allowed Gap Positions” = half) with default parameters (For ITS: 1137, nrLSU: 180, *rpb2*: 611, *tef1*: 166, *atp6*: 25 position were deleted). The software BioEdit 7.0.9 (Hall 1999) was used to manually check the aligned sequences. To examine the conflict among topologies with maximum likelihood (ML), separate single-gene analyses were conducted. Sequences were then concatenated. The ITS alignment can be found on Suppl. material 5. For the combined analyses, a partitioned mixed model was used by defining the sequences of nrLSU, *rpb2*, *tef1*, and *atp6* as four independent partitions and each gene was separately estimated by different model parameters. Maximum Likelihood (ML) and Bayesian Inference (BI) analyses were conducted on the resulting concatenated dataset.

Maximum Likelihood (ML) was performed using RAxML 8.0.14 (Stamatakis et al. 2005; Stamatakis 2006, 2014) by running 1000 bootstrap replicates under the GTRGAMMAI model (for all partitions). Bayesian Inference (BI) analysis was performed with MrBayes v3.1.2 (Ronquist and Huelsenbeck 2003) based on the best substitution models (GTR+I+G for ITS, GTR+I for nrLSU, SYM+G for *rpb2*, SYM+I+G for *tef1*, and GTR+G for *atp6*) determined by MrModeltest 2.3 (Nylander 2004). Two independent runs with four Markov chains were conducted for 10 M generations under the default settings. Average standard deviations of split frequency (ASDSF) values were far lower than 0.01 at the end of the runs. Trees were sampled every 100 generations after burn-in (25% of trees were discarded as the burn-in phase of the analyses, set up well after convergence), and a 70% majority-rule consensus tree was constructed.

Trees were visualized with TreeView32 (Page 2001). Bootstrap values (BS) ≥ 70% and Bayesian Posterior Probability values (BPP) ≥ 0.95 were considered significant (Hillis and Bull 1993; Alfaro et al. 2003).

## ﻿Results

### ﻿Phylogenetic analysis

**Figure 1.**
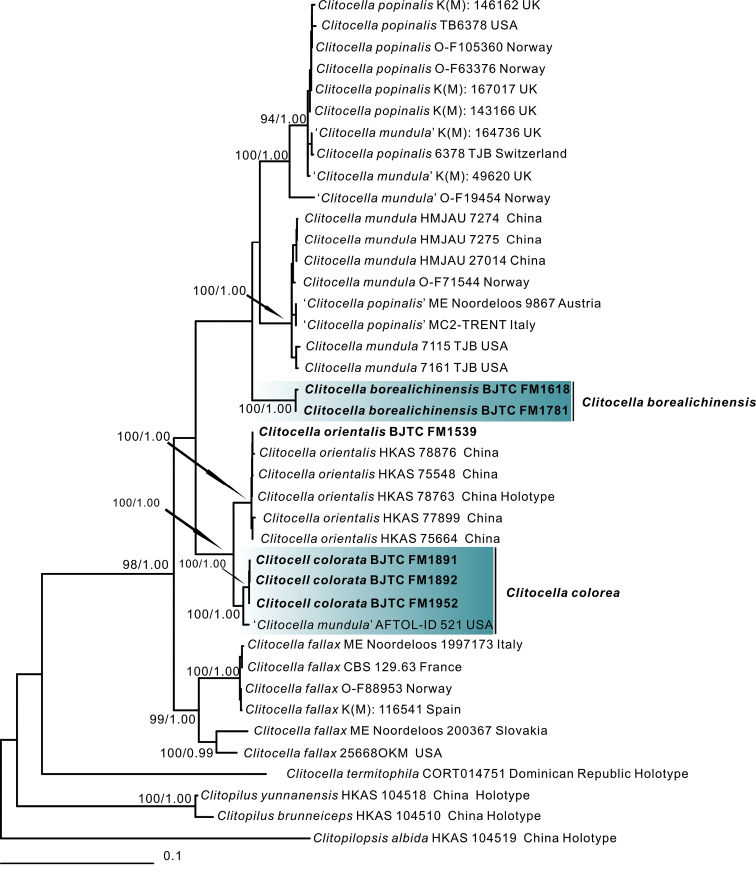
Phylogeny derived from Maximum Likelihood analysis of the combined nrLSU-*rpb2*-*tef1*-*atp6* dataset of *Clitocella* and related genera in the family Entolomataceae. *Clitopilopsisalbida* was employed to root the tree as an outgroup. Numbers representing likelihood bootstrap support (BS≥ 70%, left) and significant Bayesian posterior probability (BPP≥ 0.95, right) are indicated above the nodes. New sequences are highlighted in bold.

Twenty-eight sequences were newly generated from our six collections in this study. Two datasets, nrLSU-*rpb2*-*tef1*-*atp6* combined dataset and ITS dataset were compiled to investigate the phylogenetic position of these *Clitocella* species. For the combined dataset, the phylogenetic trees based on individual loci (including nrLSU, *rpb2*, *tef1*, *atp6*) showed the almost same major clades (Suppl. material 1–4: Figs S1–S4) as that of the combined dataset (Fig. 1). There was no strongly supported conflict between single gene phylogenies, except for the nrLSU phylogeny does not resolve *Clitocellamundula* and *C.popinalis*, while the *atp6* phylogeny does not resolve *C.orientalis* and the new species *C.colorata*. So here the combined dataset was used to infer the phylogenetic placement of *Clitocella* species. The final combined nrLSU-*rpb2*-*tef1*-*atp6* dataset contained 2963 total characters (905 from nrLSU, 599 from *rpb2*, 1010 from *tef1*, 449 from *atp6*, gaps included) and included 40 samples of 11 taxa. The topologies of ML and BI phylogenetic trees obtained in this study were practically the same, therefore only the tree inferred from the ML analysis is shown (Fig. 1). Except for the species *Clitocellatermitophila* T.J. Baroni & Angelini, members of *Clitocella* in the dataset formed a monophyletic lineage with strong support (MLB = 98%, BPP = 1.00). *Clitocellatermitophila* was sister to all other species of *Clitocella* but without strong support. Of our six collections, the sequences of a collection (BJTC FM1539) grouped in the clade *C.orientalis* S.P. Jian & Zhu L. Yang, indicating it is identity with this species. The remaining specimens fell in two strongly supported clades, one comprised of two collections was described as the new species *C.borealichinensis* and another comprised of three collections was described as the new species *C.colorata* together with a collection from USA (AFTOL-ID 521) originally labelled as *C.mundula*. *Clitocellacolorata* was sister to *C.orientalis* with strong support, implying *C.colorata* is closely related to *C.orientalis*. *Clitocellaborealichinensis* further clustered with *C.mundula* and *C.popinalis* (Fr.) Kluting, T.J. Baroni & Bergemann. One collection from Norway (O-F19454), which is labelled as *Clitocellamundula*, formed an independent clade.

**Figure 2.**
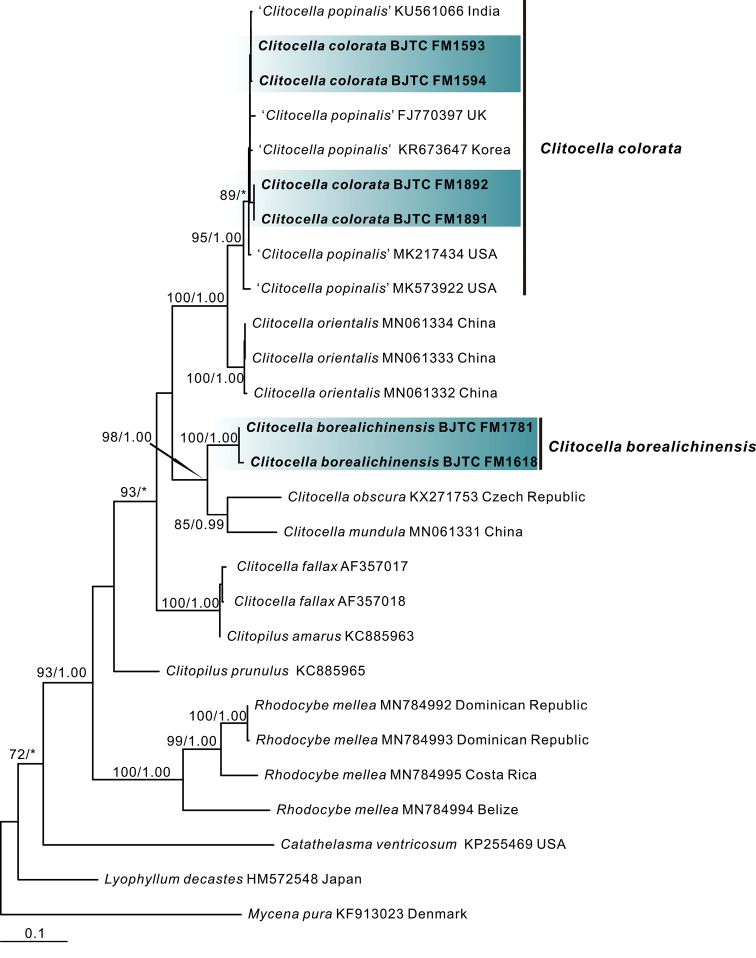
Phylogeny derived from Maximum Likelihood analysis of the ITS sequences from *Clitocella* and related genera in the family Entolomataceae. *Mycenapura* was employed to root the tree as an outgroup. Numbers representing likelihood bootstrap support (BS≥ 70%, left) and significant Bayesian posterior probability (BPP≥ 0.95, right) are indicated above the nodes. New sequences are highlighted in bold.

The ITS dataset comprised 27 samples of 11 taxa and 662 characters. The topology of phylogenetic trees based on the ITS dataset generated from ML and BI analyses were almost identical and only the tree inferred from the ML analysis is shown (Fig. 2). The sequences of the new species *C.borealichinensis* formed an independent and strong support branch, like that of multilocus phylogeny (Fig. 1), supporting it is a distinct species. The sequences of the new species *C.colorata* together with five sequences labelled as *C.popinalis* from India, South Korea, UK and USA formed an independent and strong support branch, indicating they represented a distinct species.

### ﻿Taxonomy

#### ﻿ Clitocella borealichinensis

Taxon classificationFungiAgaricalesEntolomataceae

L. Fan & N. Mao sp. nov.

13FE74D4-F341-5085-925E-6F2DF09CB4E3

 843689

Figs 3a , 4 , 6a, b

##### Etymology.

*borealichinensis*, referring to north China as the place of origin.

##### Holotype.

China. Shanxi Province, Qinshui County, Lishan Mountain, 35°36.49'N, 112°11.7'E, alt. 1150m, 26 July 2021, on the ground in broad-leaved forest dominated by *Quercus* sp., N. Mao MNM340 (BJTC FM1781).

**Figure 3.**
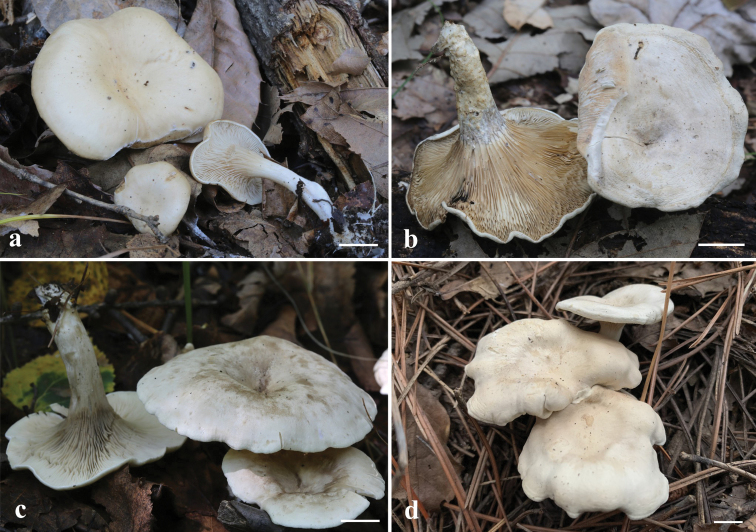
Basidiomata of *Clitocella***a***Clitocellaborealichinensis* (BJTC FM1781, holotype) **b-d***Clitocellacolorata* (**b** BJTC FM1593 **c** BJTC FM1952 **d** BJTC FM1891, holotype) Scale bars: 10 mm (**a–d**). Photos by JingZhong Cao

##### Diagnosis.

*Clitocellaborealichinensis* is characterized by its clitocyboid basidiomata, globose to subglobose, occasionally broadly ellipsoid basidiospores, the absence of hymenial cystidia and clamp connection, and usually growing in broad-leaved forests. It is most similar to *C.orientalis* but differs from it by the slightly smaller basidiospores, non-gelatinized hyphae of pileipellis and stipitipellis with pale brown to brown intracellular or parietal pigment.

**Figure 4.**
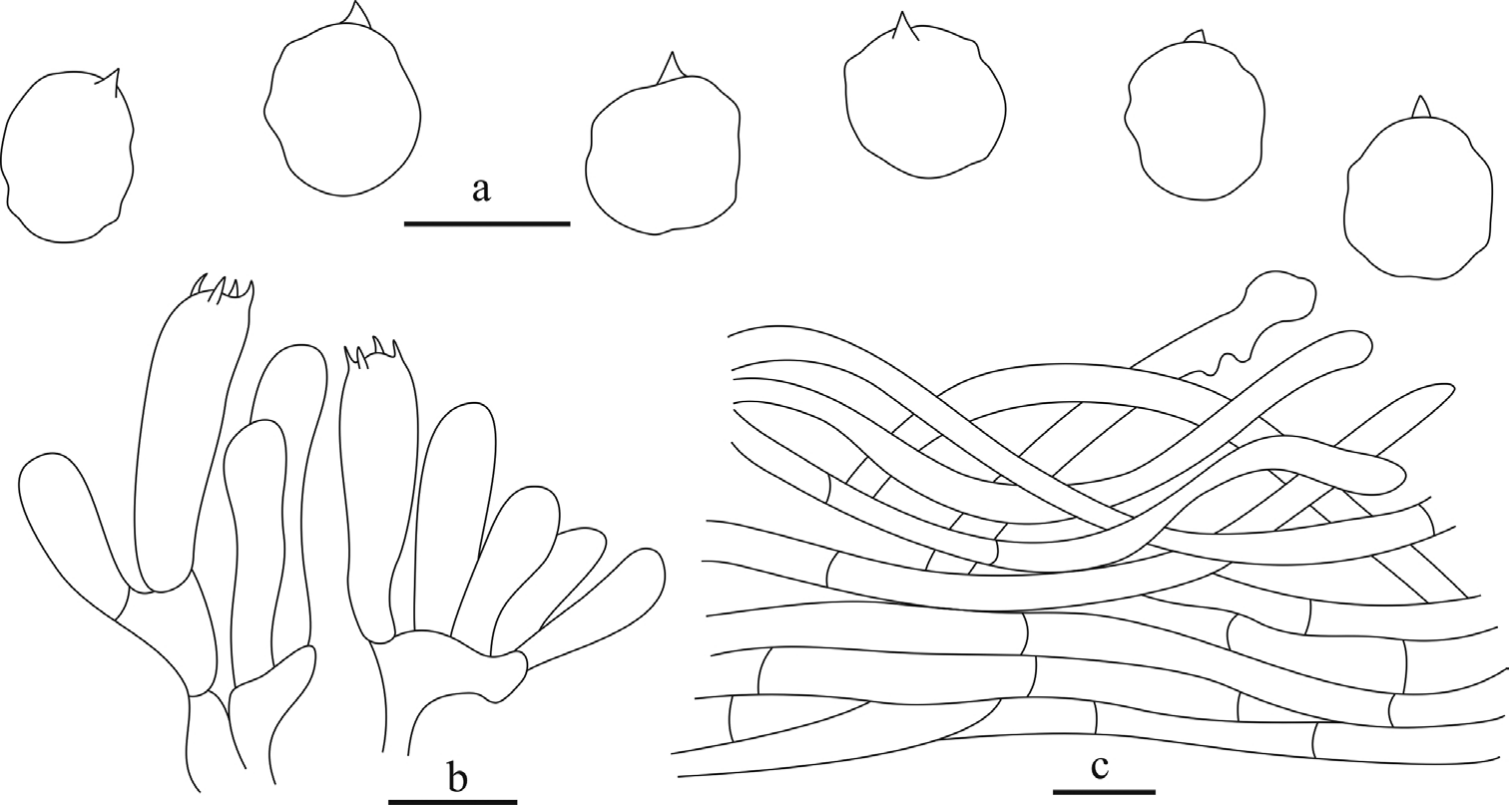
Microscopy of *Clitocellaborealichinensis***a** basidiospores **b** basidia **c** pileipellis. Scale bars: 5 μm (**a**); 10 μm (**b, c**). Drawings by Ning Mao.

##### Description.

Basidiomata clitocyboid, small to medium-sized. Pileus 13–50 mm wide, low convex to plane convex when young, then slightly depressed at center; surface smooth, grayish white (#f2f2f2) to pale white (#ffffff), yellowish white (#ffcd9a); margin incurved, non-striate; context thin pale white, 1.0–1.2 mm thick. Lamellae decurrent, grayish white (#f2f2f2), pale yellow (#fff3e7), crowded, edges smooth, thin and fragile, lamellulae numerous and concolorous with lamellae. Stipe 20–32 × 2–8 mm, central to eccentric, occasionally lateral, cylindrical to subcylindrical, equal or sometimes slightly tapering at base, pale white (#ffffff), smooth or tomentose, usually with white rhizomorphs. Odor unrecorded. Taste not recorded. Chemical color reaction: not reacting with KOH 3% at pileus of dried specimens.

Basidiospores [60/2/2] (3.8–)4–5(–5.5) × 3.8–4.5 μm, L_m_ × W_m_ = 4.61 (± 0.42) × 4.06 (± 0.18), Q = 0.95–1.25 (Q_av_ = 1.13 ± 0.10), hyaline, globose to subglobose, occasionally broadly ellipsoid in profile view, slightly angled in polar or face view with obscure minute pustules or bumps. Basidia 17–25 × 5–6(–7) μm, clavate, hyaline, four spored, rarely two spored; sterigmata 2–4 μm long. Lamellar trama more or less regular, composed of 3–8 μm wide hyaline hyphae, subhymenium consisting of filamentous hyphal segments. Lamellae edges fertile. Pleurocystidia and cheilocystidia absent. Pileipellis a cutis composed of more or less radially, loosely arranged, non-gelatinized, smooth, cylindrical hyphae, 2–6 μm wide and with pale brown to brown intracellular or parietal pigment; terminal hyphae subcylindric, narrowly clavate, occasionally irregular, 3–5 μm wide; subcutis made up of subparallel, compactly arranged, thin-walled, hyaline, smooth, cylindrical hyphae, 3–6 μm wide; pileal trama composed of interwoven, cylindrical hyphae, 2.5–10 μm wide. Stipitipellis a cutis composed of parallel, compactly arranged, thin-walled, non-gelatinized, hyaline hyphae, 2.5–6 μm wide. Stipititrama composed of interwoven, hyaline, cylindrical hyphae, 3–10 μm wide. Caulocystidia absent. Clamp connections absent.

##### Habit.

Scattered or in groups on soil in broad-leaved (*Quercus*) forest, Shanxi province, China.

##### Additional specimens examined.

**China.** Shanxi province, Xia County, alt. 970m, 7 October 2020, N. Mao MNM172 (BJTC FM1618).

##### Note.

*Clitocellaborealichinensis* is easily confused with *C.orientalis*, *C.obscura* (Pilát) Vizzini *et al.* and *C.pallescens* Silva-Filho & Cortez in morphology because they are all have white to grayish white pileus and decurrent lamellae. However, *C.orientalis* differs from *C.borealichinensis* by its viscid pileus and stipe when wet, gelatinized pileipellis and stipitipellis, and slightly larger basidiospores of (4–)4.5–6 × 4–5 μm (Jian et al. 2020). *Clitocellaobscura* produce a distinctly reddish reaction when 3% KOH is placed on the pileus surface (Baroni 1981; as *Rhodocybe*) while *C.borealichinensis* has not that kind of reaction. *Clitocellapallescens* differs *C.borealichinensis* by its pale grey to yellowish white stipe (Silva-Filho et al. 2018; Jian et al. 2020).

*Clitocellamundula* and *C.popinalis* clustered with *C.borealichinensis* in our multilocus phylogeny (Fig. 1), indicating they are phylogenetically closely related to each other. Morphologically, *C.mundula* differs from *C.borealichinensis* by its yellowish gray or brown to dark smoke gray pileus and slightly larger basidiospores of (4–)4.5–6(–6.5) × 4–5 μm (Jian et al. 2020), *C.popinalis* by its brown to grayish brown pileus, bigger basidiospores of 5.5–7–5–5.5 μm, and its pileus surface produces a reddish reaction in 3% KOH (Baroni 1981; as *Rhodocybe*). Moreover, DNA analysis also revealed that *C.borealichinensis* shared less than 91.10% similarity in *tef1* sequence with *C.mundula* and 91.20% similarity with *C.popinalis*, supporting their separation.

#### ﻿ Clitocella colorata

Taxon classificationFungiAgaricalesEntolomataceae

L. Fan & N. Mao sp. nov.

BBADB941-F329-5682-AA9B-0FCCE043FCE2

 843690

**Figure 5.**
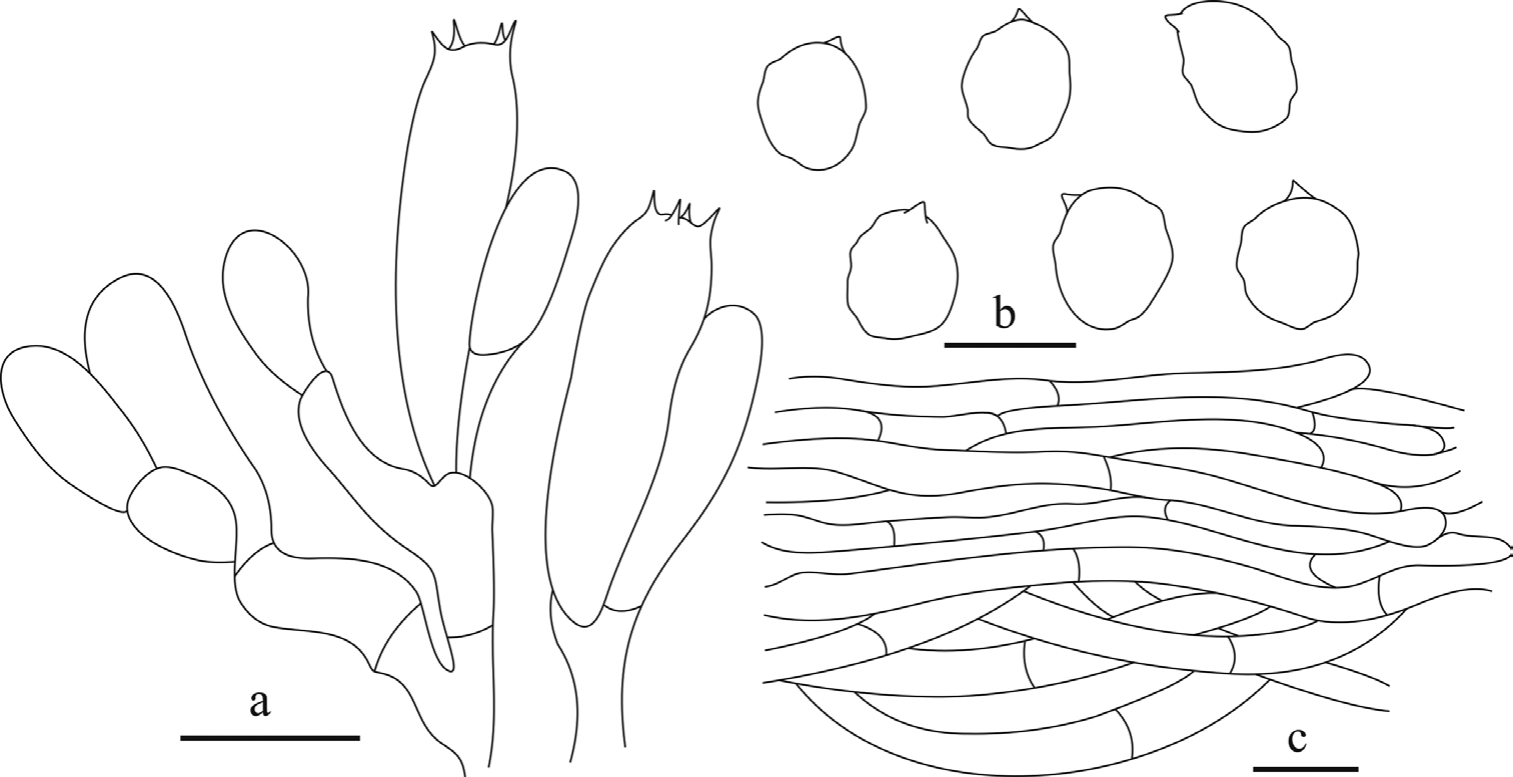
Microscopy of *Clitocellacolorata***a** basidiospores **b** basidia **c** pileipellis. Scale bars: 10 μm (**a, c**); 5 μm (**b**). Drawings by Ning Mao.

**Figure 6.**
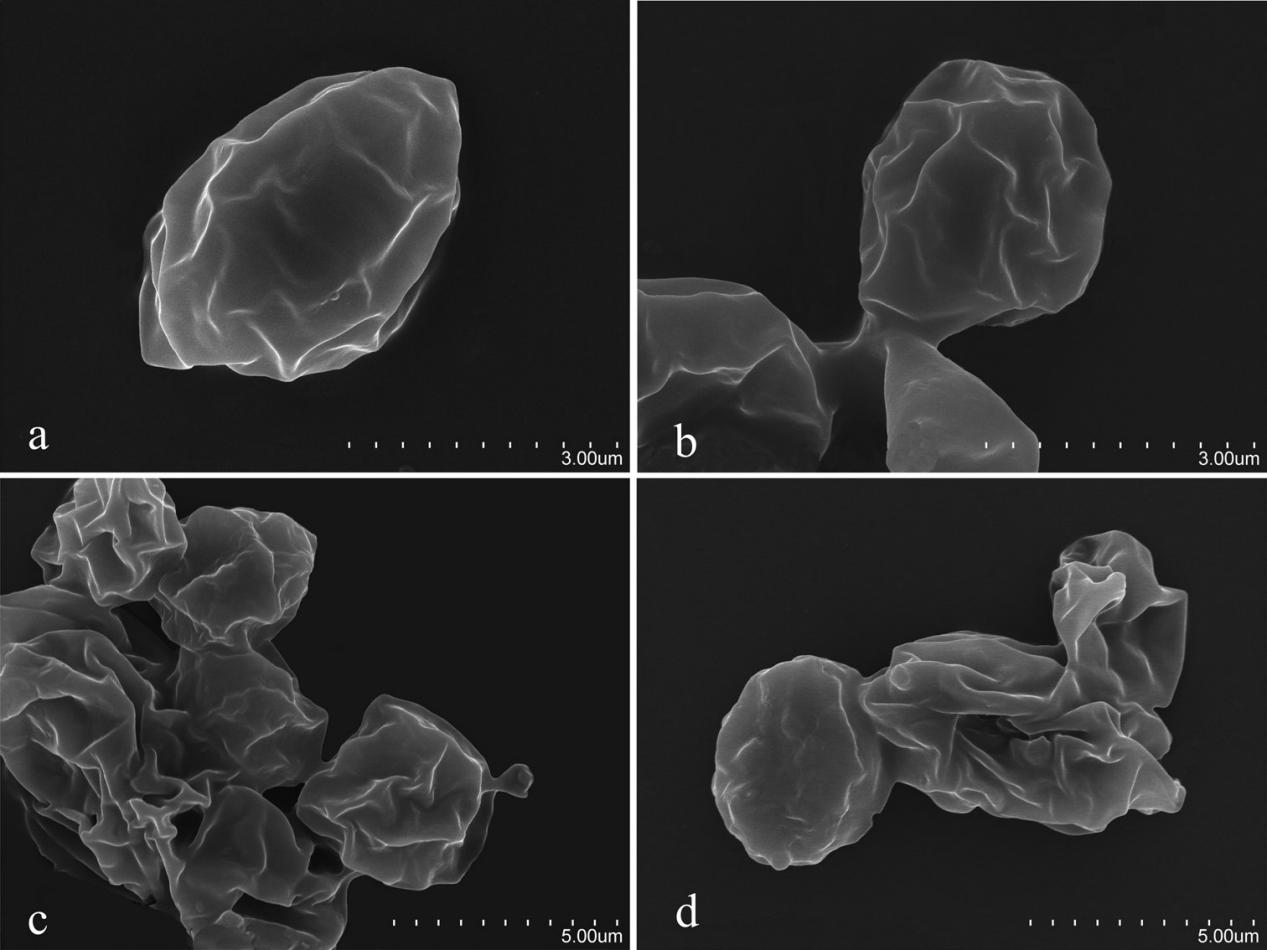
Basidiospores of species in *Clitocella*. *Clitocella* revealed by SEM**a, b***Clitocellaborealichinensis***c, d***Clitocellacolorata* Scale bars: 3 μm (**a, b**); 5 μm (**c, d**). Photos by Li Fan.

Figs 3b–d , 5 , 6c, d

##### Etymology.

*colorata*, referring to the colorful pileus.

##### Holotype.

China. Shanxi Province, Pu County, Wulushan Mountain, 36°33.2'N, 111°11.58'E, alt. 1740 m, 28 July 2021, on the ground in coniferous forest dominated by *Pinusarmandii* Franch., N. Mao MNM292 (BJTC FM1891).

##### Diagnosis.

*Clitocellacolorata* is characterized by its clitocyboid basidiomata, relatively colorful pileus (white to yellowish white, grayish white to grayish brown, pink white), globose or subglobose to broadly ellipsoid basidiospores, hyphae of pileipellis with pale yellow to yellowish brown intracellular or parietal pigment, the absence of hymenial cystidia and clamp connection. It is most similar to *C.popinalis* and *C.mundula* but differs from *C.popinalis* by its slightly smaller basidiospores, only appearing in the forest and genetic profile, and from *C.mundula* by its colorful pileus (white to yellowish white, grayish white to grayish brown, pink white).

##### Description.

Basidiomata clitocyboid, small to large. Pileus 20–62 mm wide, dry,convex to plano-convex, sometimes infundibuliform, with a shallow depression at the center; margin not striate, often enrolled or flat, sometimes slightly uplifted; surface white (#ffffff) to yellowish white (#ffffe7), grayish white (#f2f2f2) to grayish brown (#dba773), pink white (#fff3f5); context white (#ffffff) to grayish white (#f2f2f2), 1.0–1.5 mm thick. Lamellae decurrent, white (#ffffff) to yellowish white(#fff3e7), becoming yellowish brown (#e0b487) on drying, crowded, 1.0–2.0 mm deep, edges entire and concolorous, thin and fragile, lamellulae in 2–4 tiers of varying lengths. Stipe 22–42 × 4–10 mm, central, cylindrical, equal, pale white (#ffffff) to yellowish brown (#e0b487), smooth, usually with white rhizomorphs. Odor unrecorded. Taste not recorded. Chemical color reaction: pileal surface of dried samples negative with 3% KOH.

Basidiospores [100/5/2] (3.8–)4.5–5.5(–6.0) × (3.5–)4–4.8(–5.0) μm; L_m_ × W_m_ = 4.90 (± 0.44) × 4.29 (± 0.35), Q = 1.00–1.25 (Q_av_ = 1.14 ± 0.09); hyaline, globose or subglobose to broadly ellipsoid in profile view, slightly angled in polar or face view with obscure minute pustules or bumps. Basidia 20–30 × (4.5–)5–6.5 μm, clavate, hyaline, with four spored, rarely two spored; sterigmata 2–3.5 μm long. Lamellar trama composed of subparallel, hyaline, cylindrical hyphae, 2.5–6 μm wide, subhymenium consisting of filamentous hyphal segments, 2–3.5 μm wide. Lamellae edges fertile. Pleurocystidia and cheilocystidia absent. Pileipellis a cutis composed of parallel, compactly arranged, non-gelatinized, smooth, cylindrical hyphae, 2–5 μm wide, with pale yellow to yellowish brown intracellular or parietal pigment; subcutis made up of interwoven, slightly loosely arranged, hyaline, smooth, cylindrical hyphae, 3–6.5 μm wide; pileal trama composed of parallel, compactly arranged, hyaline, cylindrical hyphae, 3–10 μm wide. Stipitipellis a cutis composed of parallel, compactly arranged, thin-walled, non-gelatinized, cylindrical hyphae, 2–5 μm wide, heavily or moderately encrusted with brown pigment. Stipititrama composed of parallel, compactly arranged, hyaline, cylindrical hyphae, 3–7 μm wide. Caulocystidia absent. Clamp connections absent.

##### Habit.

Scattered or in groups on soil or rotten wood in coniferous (*Pinus*) or broad-leaved (*Quercus*) forest, Shanxi province, China.

##### Additional specimens examined.

**China.** Shanxi province, Pu County, Wulushan Mountains, alt. 1750m, 28 July 2021, N. Mao MNM293 (BJTC FM1892); Wenshui County, alt. 1760m, 30 July 2021, L. Fan CF1219 (BJTC FM1952); Xia County, alt. 931m, 6 October 2020, N. Mao MNM102 (BJTC FM1593); Xia County, alt. 931m, 6 October 2020, N. Mao MNM103 (BJTC FM1594).

##### Notes.

Morphologically, *Clitocellacolorata* is easily confused with *C.mundula* and *C.popinalis*. However, according to Baroni (1981; as *Rhodocybe*), the pileus surface in *C.mundula* and *C.popinalis* can produce a reddish reaction in 3% KOH, whereas that is not exhibited in *Clitocellacolorata*. The basidiospores of *C.popinalis*, 5.5–7 × 5–5.5 μm (Baroni 1981; Kluting et al. 2014; Jian et al. 2020), are broader and longer than those of *C.colorata* (4.5–5.5 ×4–4.8 μm). DNA analysis revealed that *C.colorata* shared less than 87.80% similarity in *tef1* sequence with *C.mundula* and 86.10% similarity with *C.popinalis*, supporting their separation. Moreover, five ITS sequences (FJ770397, KR673647, KU561066, MK217434 and MK573922) labelled “*C.popinalis*” from India, Norway, South Korea, UK and USA are probably conspecific to the new species *C.colorata* as they clustered together with *C.colorata* in ITS tree (Fig. 2) and have more than 98.4% similarity in ITS region. However, these “*C.popinalis*” collections still need more other DNA regions and detailed morphology to support this view. One collection of “*C.mundula*,” namely, AFTOLID 521 from Norway, should be re-identified *C.colorata* as it clustered together with *C.colorata* in the combined nrLSU-*rpb2*-*tef1*-*atp6* tree (Fig. 1) and have more than 98.1% similarity in *tef1* region. These showed that the new species *C.colorata* maybe have a wide geographical distribution. Although *C.orientalis* is sister to *C.colorata* with strong support, these two species have obvious differences in morphology. The pileus and stipe of *C.orientalis* are usually viscid when wet and have gelatinized pileipellis and stipitipellis. *Clitocellacolorata* has non-gelatinized pileipellis and stipitipellis, and its pileus is more colorful and darker (Jian et al. 2020). DNA analysis revealed that *C.colorata* shared less than 95.80% similarity in *tef1* sequence with *C.orientalis* and 90.20% similarity in ITS sequence. Moreover, *C.colorata* has a wider distribution range than *C.orientalis*, which is only distributed in China.

## ﻿Discussion

Three species of *Clitocella* are confirmed from Shanxi Province, north China in this study. Of them, *C.colorata* is the most commonly encountered species, which distributes across the provincial area and grows in almost all kinds of forest. *Clitocellaorientalis* and *Clitocellaborealichinensis* are probably limited in southern Shanxi province, and they usually occur in the *Quercus* spp. forests.

ITS gene is rarely used in the species classification of *Clitocella* in previous studies because it contains many ambiguous sites. In the contrast, the partial sequences of three protein-coding genes (the *atp6*, *rpb2* and *tef1*) are usually used to infer the phylogeny of *Clitocella* (Kluting et al. 2014; Baroni et al. 2020; Jian et al. 2020). However, we found that ITS, *rpb2*, and *tef1* gene tree are similar to the combined (nrLSU-*rpb2*-*tef1*-*atp6*) gene regions tree when we performed phylogenetic tree construction respectively using the ITS, nrLSU, *rpb2*, *tef1* and *atp6* gene of *Clitocella* (Fig. 2, Suppl. material 1–4: Figs S1–S4). DNA analysis also showed that the intraspecific similarity of the ITS region is ≥ 98.4% and of *tef1* gene is ≥ 98.1%, the interspecific similarity of ITS region is ≤ 96.1% and of *tef1* is ≤ 95.9% (Table 2, Table 3). But for the *rpb2* gene, the intraspecific variation of *C.mundula* is more than the interspecific variation of *C.colorata* and *C.orientalis* (Table 4). Therefore, we consider that both the ITS and *tef1* may be more effective for the classification of *Clitocella* species.

**Table 2.** Interspecific variation and intraspecific variation of ITS in *Clitocella* species.

Species	Number (n)	Intraspecific variation (%)	Interspecific variation (%)
*Clitocellacolorata*	9	< 1.6%	> 3.9%
*C.fallax*	3	< 0.3%	> 11.8%
*C.mundula*	1	–	> 6.0%
*C.borealichinensis*	2	–	> 9.6%
*C.obscura*	1	–	> 6.6%
*C.orientalis*	3	< 0.9%	> 3.9%

**Table 3.** Interspecific variation and intraspecific variation of *tef1* in *Clitocella* species.

Species	Number (n)	Intraspecific variation (%)	Interspecific variation (%)
*Clitocellacolorata*	4	< 1.9%	> 4.1%
*C.fallax* ^a^	1	–	> 9.8%
*C.fallax* ^b^	2	< 0.1%	> 9.8%
*C.mundula*	6	< 0.3%	> 7.5%
‘*C.mundula*’^c^	1	–	> 4.7%
*C.borealichinensis*	1	–	> 8.4%
*C.orientalis*	3	< 0.1%	> 4.1%
*C.popinalis*	7	–	> 4.7%

^a^ represents voucher 25668OKM; ^b^ represents voucher O-F88953, K(M): 116541; ^c^ represents voucher O-F19454

Our molecular phylogenetic analysis (Fig. 1) revealed that one Norway collection O-F19454, which is labelled as *Clitocellamundula*, formed an independent clade, and it shared less than 93.40% similarity in *tef1* sequence with other *Clitocella* species. These show that it probably represents a new species of *Clitocella*. The sequences of *Clitocellafallax* formed two or three (in *rpb2* phylogeny) independent branches in our phylogenetic analyses (Fig. 2, Suppl. material 1–4: Figs S1–S4), and the similarity between the branches is less than 90.2% in *tef1* sequence and 94.9% in *rpb2* sequence. These indicate that these specimens of *C.fallax* probably represented two or three species. The specimens of *C.fallax* should be therefore re-examined to resolve this taxonomic issue. *Clitocellatermitophila* is not clustered in the genus *Clitocella* (Fig. 1). Moreover, in the *rpb2* gene tree *C.termitophila* did not gather with *Clitocella*, *Clitopilopsis* or *Clitopilus* but formed a single branch (Suppl. material 2: Fig. S2). These indicate that *Clitocellatermitophila* probably represents a potential taxonomic position rather than the species of *Clitocella*.

**Table 4.** Interspecific variation and intraspecific variation of *rpb2* in *Clitocella* species.

Species	Number (n)	Intraspecific variation (%)	Interspecific variation (%)
*Clitocellacolorata*	4	< 0.7%	> 1.7%
*C.fallax* ^a^	1	–	> 4.0%
*C.fallax* ^b^	4	< 0.1%	> 5.1%
*C.fallax* ^c^	1	–	> 4.0%
*C.mundula*	6	< 2.1%	> 4.9%
‘*C.mundula*’^d^	1	–	> 2.2%
*C.borealichinensis*	2	–	> 5.5%
*C.orientalis*	6	< 0.5%	> 1.7%
*C.popinalis*	9	< 0.4%	> 2.2%
*C.termitophila*	1	–	> 16.9%

^a^ represents voucher 25668OKM; ^b^ represents voucher O-F88953, K(M): 116541, CBS 129.63, ME Noordeloos 1997173; ^c^ represents voucher ME Noordeloos 200367; ^d^ represents voucher O-F19454.

### ﻿Key to the species of *Clitocella*

**Table:** 

1	Basidiomata clitocyboid	**2**
–	Basidiomata pleurotoid	***C.termitophila*** ^*^ **(Baroni et al. 2020)**
2	Pileus surface gray, dark gray, pale yellow to yellowish brown, pigments present in pileipelli	**3**
–	Pileus surface almost white to pastel gray, pigments absent in pileipellis	**8**
3	Basidiospores globose to subglobose	**4**
–	Basidiospores ellipsoid	**7**
4	Pileus surface of dried samples with a positive KOH reaction	**5**
–	Pileus surface of dried samples with a negative KOH reaction	**6**
5	Occurring in grassland systems	C.popinalis*(Baroni 1981; Kluting et al. 2014; Jian et al. 2020)
–	Occurring in forested systems	C.mundula*(Baroni 1981; Kluting et al. 2014; Jian et al. 2020)
6	Pileus color with pink tinges	C.colorata*
–	Pileus color without pink tinges	C.borealichinensis*
7	Pileus color with yellow tinges, basidiospores small, 5–8 × 3.5–5.5 μm	***C.himantiigena* (Silva-Filho et al. 2018)**
–	Pileus color without yellow tinges, basidiospores large, 7–9 × 5–6 μm	***C.ammophila* (Contu 1999)**
8	Basidiospores globose to subglobose or ovatae	**9**
–	Basidiospores amygdaliform to ellipsoid	**11**
9	Basidia long, length > 40 μm	***C.nigrescens* (Maire 1945)**
–	Basidia short, length < 28 μm	**10**
10	Pileus infundibuliform to plano-convex, basidiospores 4–5 × 3–4.5 μm	***C.pallescens* (Silva-Filho et al. 2018; Jian et al. 2020)**
–	Pileus convex to plane, basidiospores (4–)4.5–6 × 4–5 μm	C.orientalis* **(Jian et al. 2020)**
11	Basidiospores small, 5–6.2 × 2.5–3.6 μm	***C.blancii* (Contu 2009)**
–	Basidiospores large, 6.5–8 × 4–5 μm	C.fallax* **(Jian et al. 2020)**

## Supplementary Material

XML Treatment for Clitocella borealichinensis

XML Treatment for Clitocella colorata

## Footnotes

Indicates the presence of molecular data.

## Supplementary materials

Supplementary material 1 Figure S1 Data type JPG file Explanation note Phylogeny derived from Maximum Likelihood analysis of the *nrLSU* dataset of *Clitocella* and related genera in the family Entolomataceae. The bootstrap frequencies (> 70%) is shown on the supported branches. New species are highlighted in red. https://binary.pensoft.net/file/672117This dataset is made available under the Open Database License (http://opendatacommons.org/licenses/odbl/1.0/). The Open Database License (ODbL) is a license agreement intended to allow users to freely share, modify, and use this Dataset while maintaining this same freedom for others, provided that the original source and author(s) are credited.Ning Mao, Jing-Chong Lv, Yu-Yan Xu, Tao-Yu Zhao, Li Fan

Supplementary material 2 Figure S2 Data type JPG file Explanation note Phylogeny derived from Maximum Likelihood analysis of the *rpb2* dataset of *Clitocella* and related genera in the family Entolomataceae. The bootstrap frequencies (> 70%) is shown on the supported branches. New species are highlighted in red. https://binary.pensoft.net/file/672118This dataset is made available under the Open Database License (http://opendatacommons.org/licenses/odbl/1.0/). The Open Database License (ODbL) is a license agreement intended to allow users to freely share, modify, and use this Dataset while maintaining this same freedom for others, provided that the original source and author(s) are credited.Ning Mao, Jing-Chong Lv, Yu-Yan Xu, Tao-Yu Zhao, Li Fan

Supplementary material 3 Figure S3 Data type JPG file Explanation note Phylogeny derived from Maximum Likelihood analysis of the *tef1* dataset of *Clitocella* and related genera in the family Entolomataceae. The bootstrap frequencies (> 70%) is shown on the supported branches. New species are highlighted in red. https://binary.pensoft.net/file/672119This dataset is made available under the Open Database License (http://opendatacommons.org/licenses/odbl/1.0/). The Open Database License (ODbL) is a license agreement intended to allow users to freely share, modify, and use this Dataset while maintaining this same freedom for others, provided that the original source and author(s) are credited.Ning Mao, Jing-Chong Lv, Yu-Yan Xu, Tao-Yu Zhao, Li Fan

Supplementary material 4 Figure S4 Data type JPG file Explanation note Phylogeny derived from Maximum Likelihood analysis of the *atp6* dataset of *Clitocella* and related genera in the family Entolomataceae. The bootstrap frequencies (> 70%) is shown on the supported branches. New species are highlighted in red. https://binary.pensoft.net/file/672120This dataset is made available under the Open Database License (http://opendatacommons.org/licenses/odbl/1.0/). The Open Database License (ODbL) is a license agreement intended to allow users to freely share, modify, and use this Dataset while maintaining this same freedom for others, provided that the original source and author(s) are credited.Ning Mao, Jing-Chong Lv, Yu-Yan Xu, Tao-Yu Zhao, Li Fan

Supplementary material 5 ITS alignment Data type PHY file Explanation note The ITS dataset comprised 27 samples of 11 taxa and 662 characters. https://binary.pensoft.net/file/672121This dataset is made available under the Open Database License (http://opendatacommons.org/licenses/odbl/1.0/). The Open Database License (ODbL) is a license agreement intended to allow users to freely share, modify, and use this Dataset while maintaining this same freedom for others, provided that the original source and author(s) are credited.Ning Mao, Jing-Chong Lv, Yu-Yan Xu, Tao-Yu Zhao, Li Fan
