# Supplementary figures and images for: ﻿Two new Clitocella species from North China revealed by phylogenetic analyses and morphological characters
Source: MycoKeys. 2022 Apr 13;88:151–70. doi: 10.3897/mycokeys.88.80068 (PMC9021152; doi:10.3897/mycokeys.88.80068)

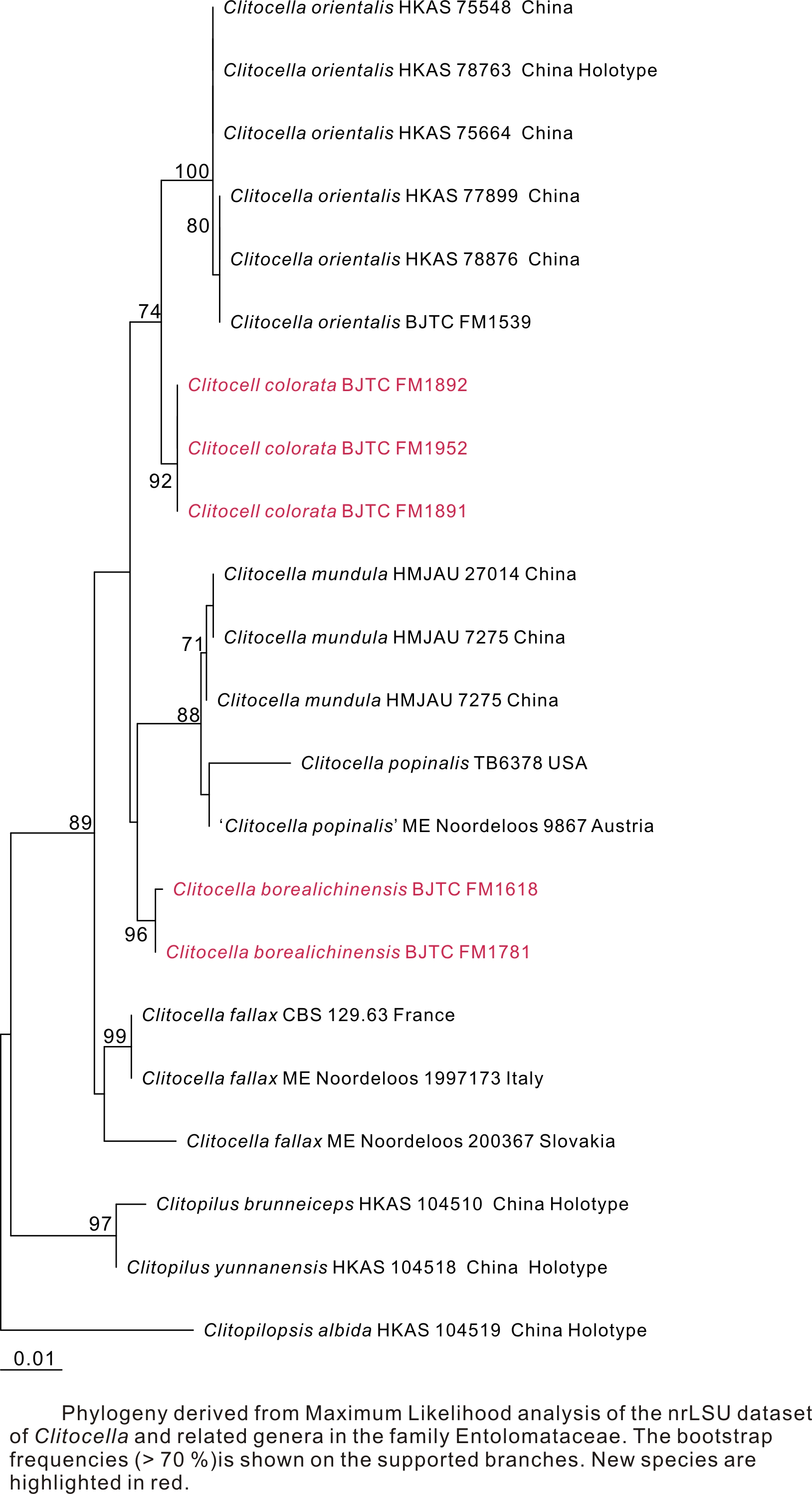

Supplement: Supplementary material 1 — Figure S1 [file mycokeys-88-151-s001.jpg]

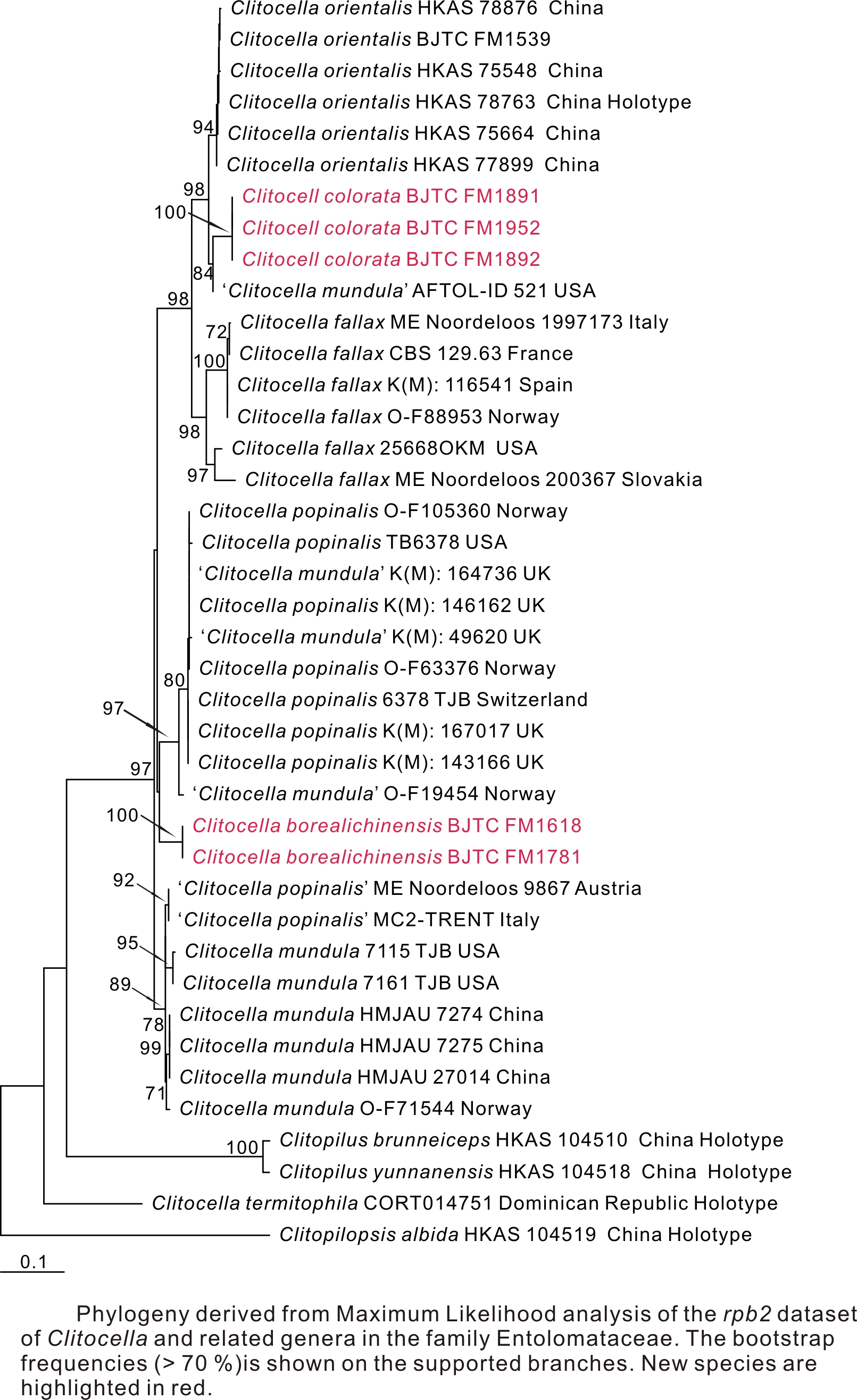

Supplement: Supplementary material 2 — Figure S2 [file mycokeys-88-151-s002.jpg]

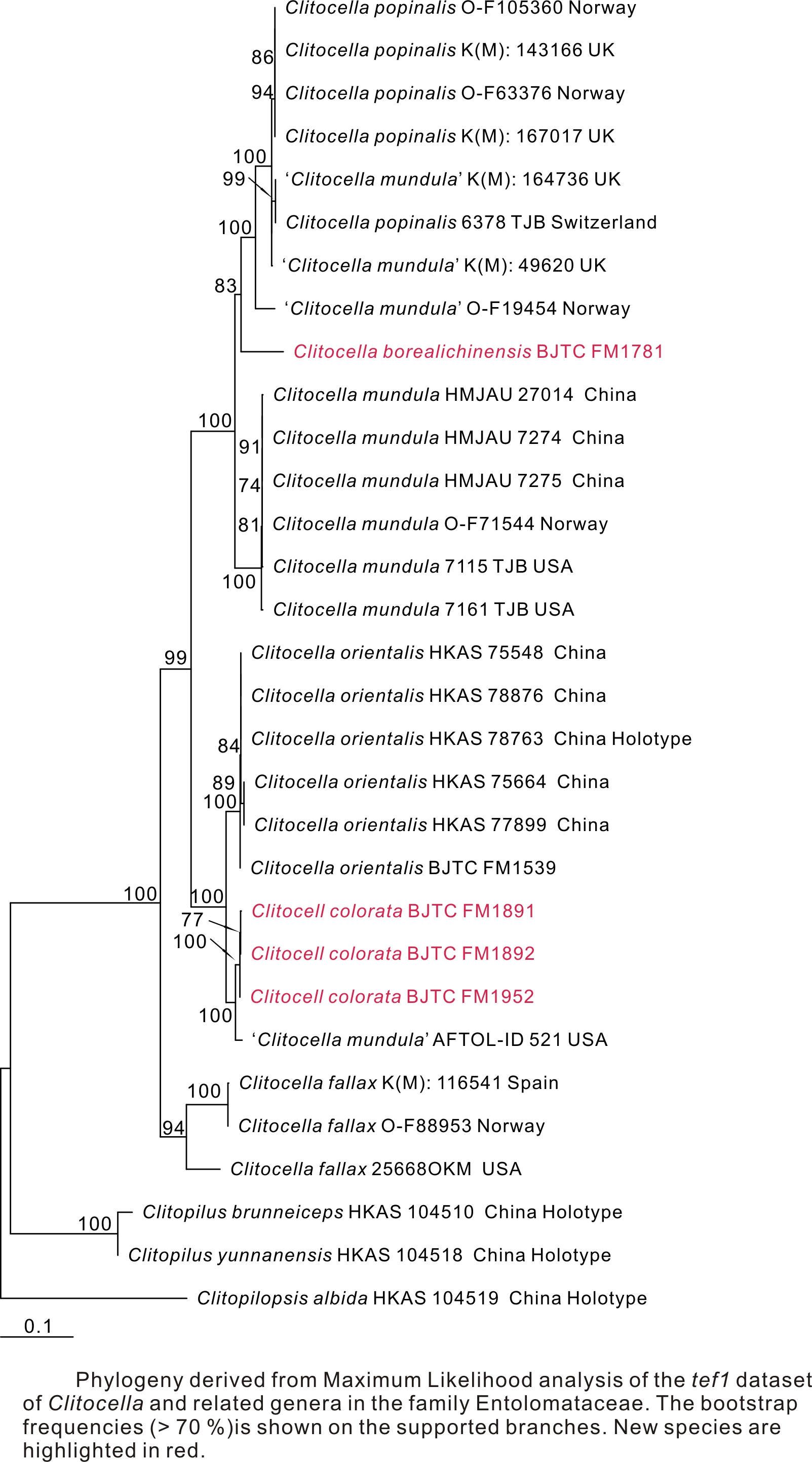

Supplement: Supplementary material 3 — Figure S3 [file mycokeys-88-151-s003.jpg]

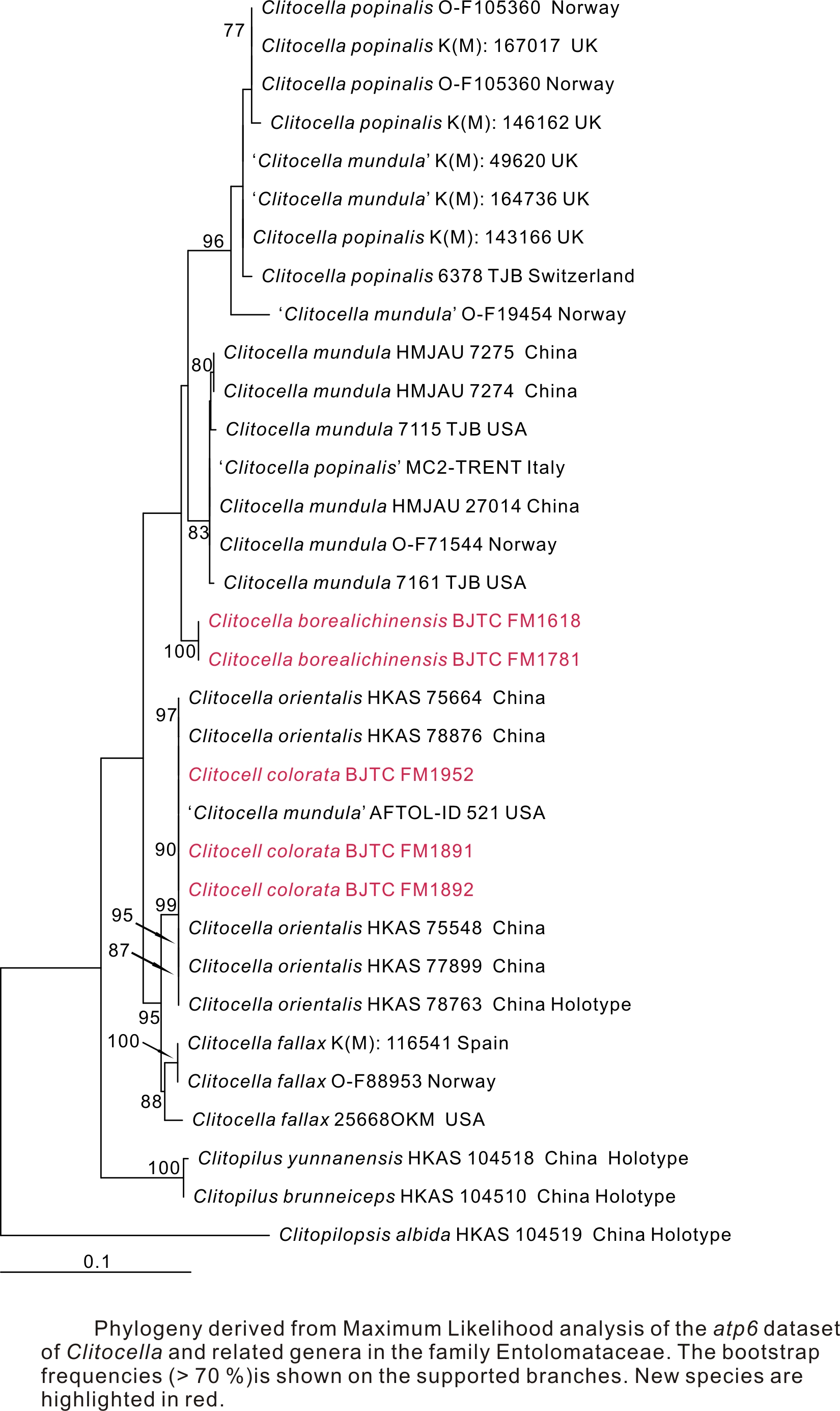

Supplement: Supplementary material 4 — Figure S4 [file mycokeys-88-151-s004.jpg]
